# Study on the pollution law of complex working conditions in sand-filled fracture well washing operation

**DOI:** 10.1371/journal.pone.0343870

**Published:** 2026-06-08

**Authors:** Wei Shi, Shifeng Zhang, Dejun Li, Kang Zhang, Xingbang Meng, Qi Yang, Yan Zhuang

**Affiliations:** 1 CNPC Tubular Goods Research Institute, Xi′an, Shaanxi, China; 2 School of Petroleum and Natural Gas Engineering, Changzhou University, Changzhou, China; 3 The 4th oil Production Plant, Changqing oilfield, Jingbian, China; Dawood University of Engineering and Technology, PAKISTAN

## Abstract

Aiming at the problem of wellbore leakage in the process of sand washing in horizontal wells, this study systematically analyzed the influence of temperature and pressure on the plugging behavior of flushing fluid through the combination of numerical simulation and experimental experiment, and carried out field application. Based on CT technology, the fracture pore structure was reconstructed, and the particle gradation of the well flushing fluid was optimized by combining the Vickers criterion. The particle-fluid interaction model was established by CFD-DEM coupling method. The experimental and simulation results show that the increase of pressure will enhance the sand carrying capacity of fluid, which will lead to the increase of particle invasion depth and aggravate the risk of deep pollution. When the temperature increases, the shallow retention capacity of the particles is enhanced by promoting the thermal expansion of the particles and reducing the viscosity of the well flushing fluid, and the invasion depth is significantly reduced, forming an efficient shallow plugging. The research reveals the law of pressure and temperature on pollution behavior, and provides a theoretical basis for particle system optimization and process parameter design of well washing operation under complex working conditions. The field test shows that the well washing fluid suitable for the formation temperature is optimized, and the construction displacement is reasonably controlled, which can effectively reduce the leakage in the sand washing process and shorten the production recovery period

## 1. Introduction

In the process of drilling operation, lost circulation, as a common and dangerous drilling accident, refers to the phenomenon that the working fluid (such as cement slurry, etc.) in the wellbore leaks into the formation cracks or pores [1,2]. When drilling in fractured and vuggy formations, lost circulation is particularly frequent, which has become one of the key factors that seriously restrict drilling safety [3–5]. The causes of lost circulation are complex and diverse, including the large pores in the formation itself, the improper measures taken in the drilling process, and the failure to achieve a good match between the formation pressure and the wellbore pressure [6]. Once a malignant lost circulation accident occurs, it will not only cause interference and obstruction to the normal drilling operation process, but also lead to a series of bad consequences such as wellbore instability and blowout, which poses a great threat to drilling safety [7–9].

In 1977, Abrams et al. first proposed the ’ 1 / 3 bridging rule ‘, which can effectively reduce the degree of reservoir damage when the particle size of the plugging particle is greater than or equal to one-third of the average pore diameter of the formation, but the criterion does not optimize the particle size distribution [10]. By applying the ideal filling theory and using the graphical method, Dick et al. studied how to select and optimize the combination of bridging particles in drilling fluid and plugging to achieve effective plugging of formations with different permeability, reduce filtrate and solid phase invasion, and improve well production efficiency [11]. Based on the ’ ideal filling theory ‘, Hands et al. proposed the D90 rule which is easier to operate on site. The rule points out that the d90 value on the cumulative distribution curve of the particle size of the temporary plugging agent should be consistent with the maximum pore size or maximum fracture width of the reservoir [12]. Based on the D90 rule, Vickers et al. proposed the Vickers criterion, and studied how to accurately match the particle size distribution of bridging particles in drilling fluid with the pore throat size distribution of reservoir, so as to achieve more efficient plugging effect, reduce formation damage during drilling, and significantly improve the production performance of oil wells [13].

The influence of different working conditions on the plugging effect is reflected in many aspects. Song Lifang et al. found that high temperature will affect the plugging material. For this reason, a flexible particle loss circulation material for drilling fluid was developed, which can maintain good toughness and expansion characteristics under high temperature environment [14]. Alsaba Mortadha et al. found that the lost circulation materials particles with different shapes and sizes may have different expansion and deformation behaviors at high temperatures, thus affecting their ability to seal cracks [15]. Wang Xiaoyu et al. predicted the distribution of particles in fractures by comprehensively considering factors such as particle size and shape. It is found that the particles with too large volume will accumulate at the entrance of the fracture, while the particles with smaller volume will form a plugging in the fracture area [16]. Qu Hai et al. verified each other through experiments and simulations, and deeply explored the effects of particle density, fluid velocity and viscosity on particle migration [17]. Dai Jianjun et al. found that the volume, shape and compressibility of particles will increase the probability of blockage [18].

In order to solve the problem of wellbore leakage in the process of sand washing in horizontal wells, this study systematically studied the plugging effect of well washing fluid under different working conditions based on the plugging theory with good compatibility with strata. In the optimization of washing fluid particle system, Shi wei et al. compared the applicability of Vickers criterion, D90 rule and ideal filling theory by using CFD-DEM method. It is concluded that the Vickers criterion has good rationality and effectiveness in the configuration of plugging material particle size [19], so the Vickers criterion is finally selected as the theoretical basis for the parameter setting of washing fluid particles. In order to further accurately characterize the pore characteristics of the sand-filled fracture area, the pore structure of the fracture area was reconstructed by means of CT technology, and the parameters of the particles in the well flushing fluid were determined based on the pore size distribution data. Finally, the influence of temperature and pressure on the well washing process is systematically analyzed by the combination of numerical simulation and indoor simulation experiment. The purpose of this study is to reveal the formation mechanism of reservoir pollution under complex working conditions, so as to provide theoretical support and technical reference for the optimization of on-site well washing operation.

## 2. Experiments and basic models

### 2.1. Experimental samples and procedures

#### 2.1.1. Experimental samples.

**Fig 1:**
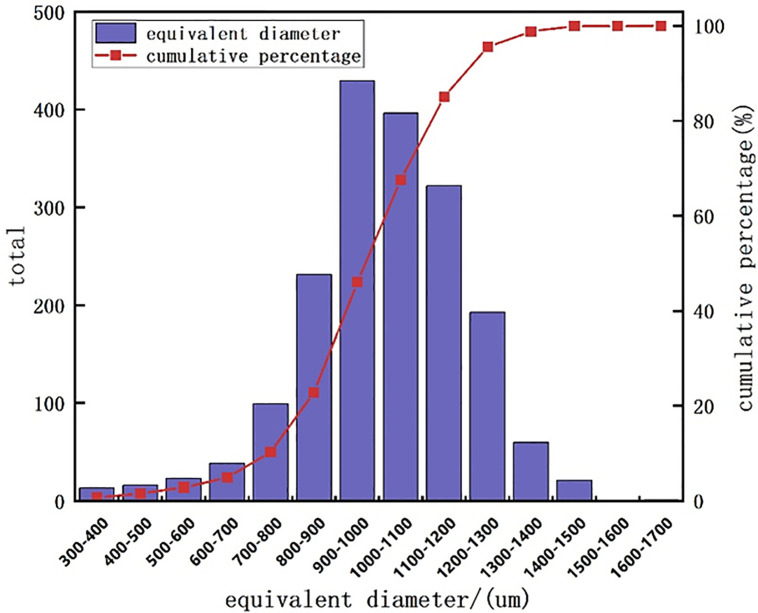
Pore-size distribution in the proppant-packed fracture zone [19].

The experiment employed a standard steel core with an artificial fracture as the test sample. The core has a diameter of 2.5 cm and a height of 5.0 cm. A fracture with a width of 5 mm and a length of 17 mm was machined into the core and packed with quartz sand in the size range of 20–40 mesh. The pore size distribution characteristics of the fracture area were obtained by CT technology, and the results are shown in Fig 1. The porosity of the filled area is 34.5%, the maximum pore diameter is 1691.7μm and the average pore diameter is 1013.47μm [19]. The base fluid for the experiments was fresh water. For every liter of water, 3% bentonite was first added and allowed to hydrate for 24 hours. Additionally, 0.5% polyacrylamide (PAM) and 6% plugging agent were incorporated. The plugging agent was composed of four particle-size fractions, each accounting for 25% by weight: 8–26 mesh, 26–45 mesh, 45–100 mesh, and 100–200 mesh.

#### 2.1.2. Experimental procedure.

**Fig 2:**
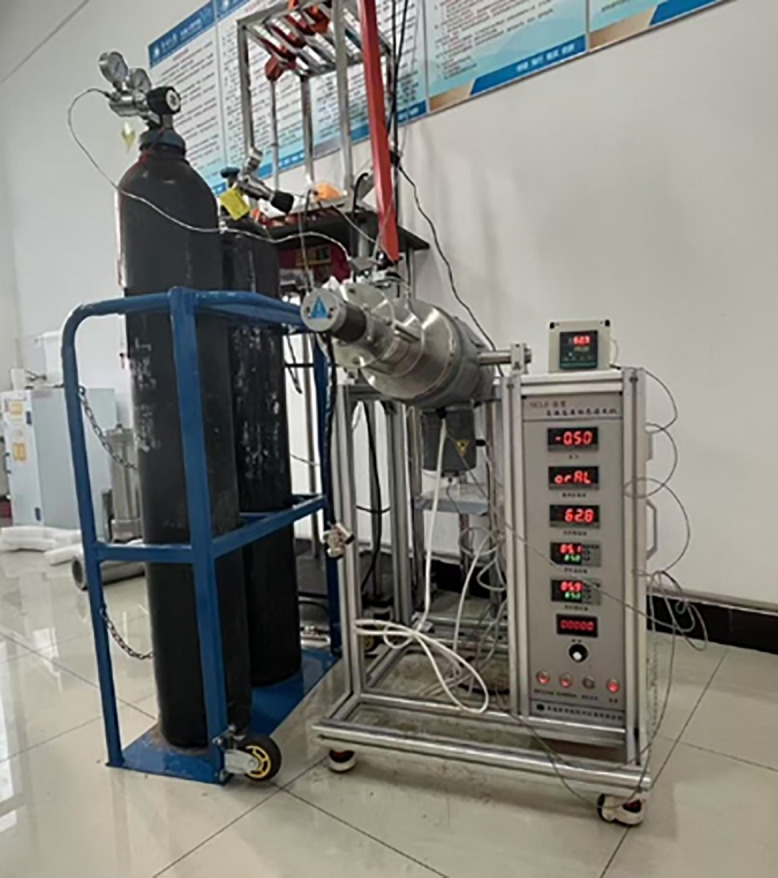
High-temperature high-pressure dynamic fluid loss apparatus [19].

In order to explore the mechanism of different working conditions on the pollution process of sand filling cracks, this experiment carried out dynamic pollution simulation experiments based on pressure and temperature. In this study, the high temperature and high pressure dynamic filtration instrument (Fig 2) was used to carry out the experiment. During the experiment, the core was placed in the core holder, the confining pressure was applied to the device through the nitrogen bottle, and the pressure value required for the experiment was accurately set by connecting the top of the device to the gas cylinder; at the same time, the whole system is heated by the heating sleeve around the device, and the display instrument equipped on the right side of the device can present the temperature data in real time, so as to realize the accurate control and monitoring of the temperature.

(1) Dynamic pollution experiments under different pressures

Five groups of experiments were set up. Under the condition of constant confining pressure of 7 MPa, the displacement pressure was set to 2,3,4 and 5 MPa in turn, and other conditions were kept consistent to simulate the pollution process under different pressure conditions.

(2) Dynamic pollution experiments at different temperatures

Five groups of experiments were set up, and other conditions were kept unchanged. The experimental temperatures were set to 45 °C, 65 °C, 85 °C and 105 °C in turn to simulate the pollution process under different temperature conditions.

### 2.2. Numerical simulation

According to the parameters obtained from the experimental results, the basic model for simulation calculation is constructed, and the simulation calculation of different working conditions is carried out.

#### 2.2.1. Numerical simulation methodology.

In this study, the method of computational fluid dynamics and discrete element (CFD-DEM) coupling is used. In the fluid, it is regarded as free flow and solved by N-S equation. The continuity and momentum conservation equations are as follows [20,21]:


$$\begin{array}{c} \frac{\partial }{\partial t}\left(\epsilon_{f}\rho_{f}\right)+\nabla \cdot \left(\epsilon_{f}\rho_{f}u_{f}\right)=\text{0} \end{array}$$ (1)

$$\begin{array}{c} \frac{\partial }{\partial t}\left(\epsilon_{f}\rho_{f}\right)+\nabla \cdot \left(\epsilon_{f}\rho_{f}u_{f}\right)=-\epsilon_{f}\nabla p+\nabla \cdot \left(\epsilon_{f}\tau \right)+\epsilon_{f}\rho_{f}g+\overset{p}{\underset{f}{f}} \end{array}$$ (2)

In the equation, $\epsilon_{f}$ denotes the volume fraction of the fluid; $\rho_{f}$ is the fluid density (unit: kg/m³); $u_{f}$ is the fluid velocity (unit: m/s); p is the fluid pressure (unit: Pa); $\overset{p}{\underset{f}{f}}$ represents the momentum source term, i.e., the force exerted by particles on the fluid within each fluid control volume (unit: N/m³); τ is the stress tensor of the fluid; g is the gravitational acceleration (unit: m/s²).

Based on the mass conservation equation and the momentum conservation equation, the fluid flow can be described as:


$$\begin{array}{c} \frac{\partial }{\partial t}\left(\rho \vec{v}\right)+\nabla \cdot \left(\rho \vec{v}\vec{v}\right)=-\nabla p+\nabla \cdot \left(\overset{―}{\overset{―}{\tau }}\right)+\rho \vec{g}+\vec{F} \end{array}$$ (3)

In the equation, ρ is the fluid density (unit: kg/m³); $\vec{v}$ is the fluid velocity (unit: m/s); p is the static pressure (unit: Pa); $\overset{―}{\overset{―}{\tau }}$ is the stress tensor; $\vec{F}$ represents the body force and external forces (unit: N).

For Reynolds number $R_{e}$[22]:


$$\begin{array}{c} \frac{d\vec{u_{p}}}{dt}=F_{D}\left(\vec{u}-\vec{u_{p}}\right)+\frac{\vec{g}\left(\rho_{p}-\rho \right)}{\rho_{p}}+\vec{F} \end{array}$$ (4)

$$\begin{array}{c} R_{e}=\frac{\rho d_{p}\left|\vec{u_{p}}-\vec{u}\right|}{\mu } \end{array}$$ (5)

In the equation, $F_{D}$ is the additional acceleration applied to the particle (unit: m/s²); $\rho_{p}$ is the density of nanoparticles (unit: g/cm³); $\vec{u_{p}}$ is the particle concentration (unit: m/s); μ is the phase velocity of the fluid (unit: m/s); $\vec{F}$ is the drag force per unit mass of the particle (unit: N); $\vec{u}$ is the dynamic viscosity of the fluid phase (unit: mPa·s); $d_{p}$ is the diameter of the nanoparticle (unit: m).

Particle trajectory and particle velocity can be described as:


$$\begin{array}{c} \frac{du_{p}}{dt}=a+\frac{\text{1}}{\tau_{p}}\left(u-u_{p}\right) \end{array}$$ (6)

In the equation, a is the acceleration due to factors other than particle drag (unit: m/s²).

#### 2.2.2. Basic model.

**Fig 3:**
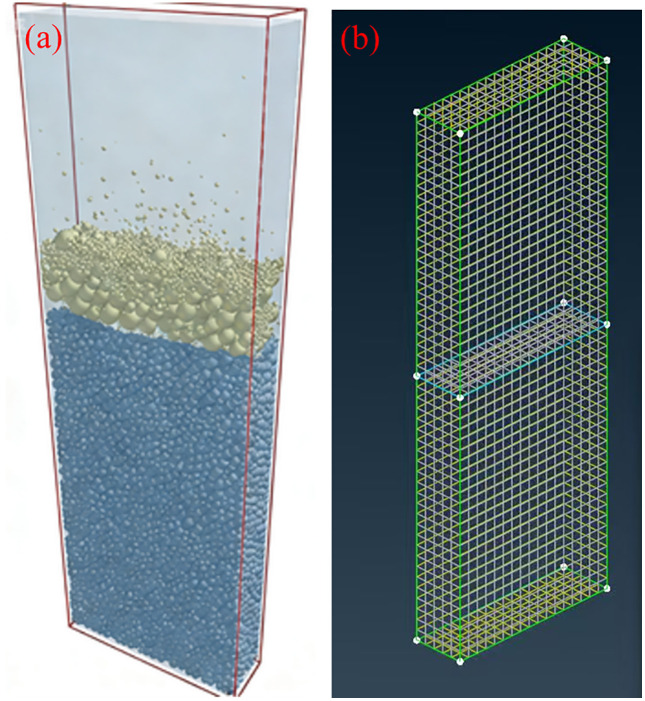
Basic model. (a) shows the initial state of the model in the CFD simulation. (b) represents the mesh generation of the model.

The structure of the particle system model constructed is shown in Fig 3. The model is a vertical transparent cuboid container with a length, width and height of 17 mm, 5 mm and 50 mm, respectively, and the mesh size is 1 mm * 1 mm * 1 mm. The model is filled with two layers of particles with different colors: the upper layer is yellow-green particles (particle 1), the lower layer is blue particles (particle 2), and the whole is clearly layered. The filling layer composed of blue particles is used to simulate the sand filling crack area, and its material mechanical properties, material properties and random particle size distribution of each particle are set respectively.

According to previous studies, the Vickers criterion has good compatibility with the target formation. In this study, the Vickers criterion model was used as a plugging model for simulation analysis.

In the process of particle migration, the particle size will change the stress state and trajectory of the particles in the fracture, and then affect the whole migration process. Therefore, it is necessary to set the particle size [23,24]. According to Vickers criterion, d25 particles = 1/7D pore throat diameter, d50 particles ≈ 1/3D pore throat diameter, d75 particles < 2/3D pore throat diameter. The particle size combinations of the particle system were 0.05625 mm, 0.1775 mm, 0.315 mm and 0.765 mm, respectively. The working mechanism of the model is that the fluid carrying small particles impacts the pore space simulated by the filling layer particles from top to bottom, so as to realize the plugging effect. On the basis of this structure, the subsequent simulation will adjust the relevant parameters according to different working conditions, and carry out numerical calculation under complex working conditions.

#### 2.2.3. Simulation steps.

In order to systematically study the influence of different working conditions on the pollution process of sand filling cracks, the numerical simulation method was used to simulate and analyze the pollution process under the conditions of pressure and temperature changes. The specific steps are as follows:

(1) Simulation of different pressure pollution process

Based on the constructed particle-based model, three groups of different displacement pressures were set, which were 0 MPa, 0.5 MPa and 1 MPa, respectively, to simulate the interaction between fluid and particle system under different pressure gradients. Finally, the simulation results under various pressure conditions are sorted out and analyzed.

(2) Simulation of different temperature pollution process

On the basis of the same model, the verification of the basic model is completed first. Then, the temperature was set to 60 °C, 80 °C and 100 °C respectively to simulate the influence of temperature change on the migration and plugging behavior of well washing fluid in sand-filled fractures. Finally, the simulation results at different temperatures are summarized and compared.

The parameter settings of fluid inflow, outflow, temperature and pressure are shown in Table 1–4.

**Table 1:** Parameter setting of fluid inlet boundary conditions.

Parameter	Value
Velocity Specification Method	Magnitude, Normal to Boundary
Reference Frame	Absolute
Velocity Magnitude (m/s)	1(constant)
Supersonic/Initial Gauge Pressure (pascal)	0(constant)
Specification Method	Intensity and Viscosity Ratio
Turbulent Intensity (%)	5
Turbulent Viscosity Ratio	10

**Table 2:** Parameter settings of fluid outlet boundary conditions.

Parameter	Value
Backflow Reference Frame	Absolute
Gauge Pressure (pascal)	0(constant)
Pressure Profile Multiplier	1
Backflow Direction Specification Method	Normal to Boundary
Backflow Pressure Specification	Total Pressure
Specification Method	Intensity and Viscosity Ratio
Backflow Turbulent Intensity (%)	5
Backflow Turbulent Viscosity Ratio	10

**Table 3:** Fluid temperature boundary condition parameter setting.

Parameter	Value	Value	Value
Poisson’s Ratio (ν)	0.28	0.33	0.35
Solids Density (ρ)	2450 kg/m³	2425 kg/m³	2400 kg/m³
Shear Modulus (G)	8e + 07 Pa	6.5e + 07 Pa	5e + 07 Pa
Young’s Modulus (E)	2.048e + 08 Pa	1.729e + 08 Pa	1.35 e + 08 Pa
Work Function	0 eV	0 eV	0 eV
Coefficient of Restitution	0.5	0.5	0.5
Coefficient of Static Friction	0.5	0.5	0.5
Coefficient of Rolling Friction	0.01	0.01	0.01

**Table 4:** Parameter setting of fluid pressure boundary conditions.

Parameter	Value
Velocity Specification Method:	Magnitude, Normal to Boundary
Reference Frame	Absolute
Velocity Magnitude (m/s)	1(constant)
Supersonic/Initial Gauge Pressure (pascal)	0，500000，1000000
Specification Method	Intensity and Viscosity Ratio
Turbulent Intensity (%)	5
Turbulent Viscosity Ratio	10

## 3. Results and discussion

### 3.1. Simulation Results

#### 3.1.1. Simulation results under different pressures.

**Fig 4:**
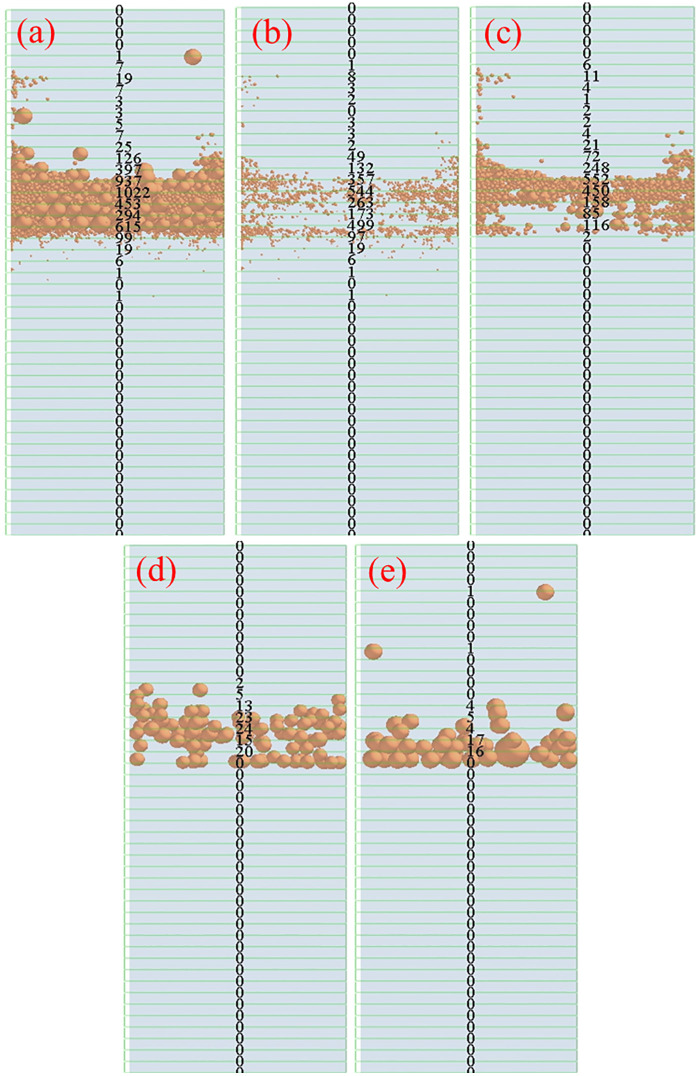
The results of particle migration under pressure of 0 MPa. (a) to (e) show the infiltration depths of the total particles and particles 1,2,3,4, respectively.

**Fig 5:**
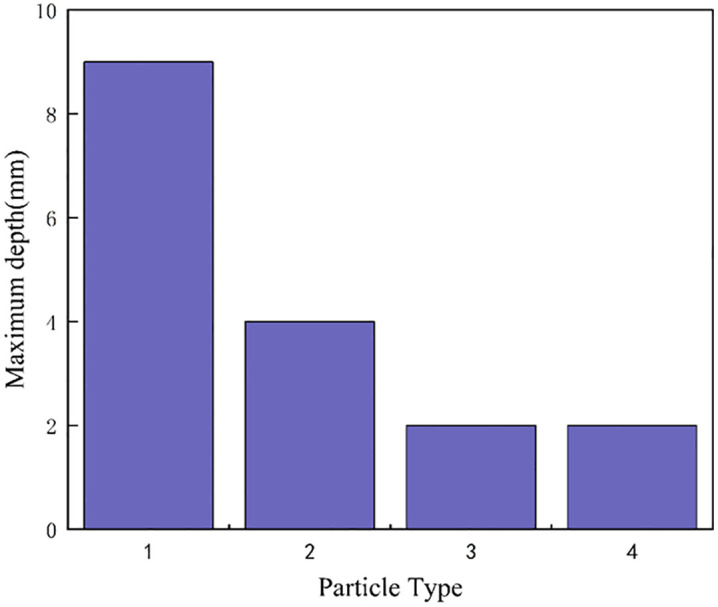
Depth of particle infiltration under pressure of 0 MPa.

In order to explore the pollution mechanism under the complex working conditions of sand-filled fracture well washing operation, the related research on the fluid parameters under the setting of 0MPa, 0.5MPa, 1MPa system pressure and 1m / s fixed flow rate was carried out. The infiltration process of particles was simulated by setting solid particles, and the dynamic pollution process was numerically simulated based on Vickers criterion model. The particle simulation results under pressure of 0MPa are shown in Fig 4 and Fig 5.

In Fig 4, the values in the figure represent the number of particles in each layer, starting from 1022 in Fig 4a, and each layer below represents a depth of 1 mm. From the perspective of infiltration depth, the infiltration depth of particle 1 reached 9 mm, indicating that the particles with smaller particle size showed strong mobility in the cracks. Due to its small particle size, its movement in the fluid is relatively less hindered, and it can penetrate into the formation with the fluid more deeply. It is not easy to form effective plugging in the shallow layer and is more dispersed in the deeper layer. The infiltration depth of particle 2 is 4 mm, and its infiltration capacity is significantly weaker than that of particle 1. This means that with the increase of particle size, the migration ability of particles in fractures is limited to a certain extent, and they begin to accumulate gradually in the shallow layer, forming a certain degree of plugging in the shallow layer, but the plugging effect may be affected by the further infiltration of particles. The infiltration depth of particles 3 and 4 is 2 mm.Due to their large particle size, they are subjected to greater resistance when moving in the fluid, and the interaction with the surface of the sand-filled fracture is stronger. It is almost difficult to invade into the formation and gather more near the surface.

**Fig 6:**
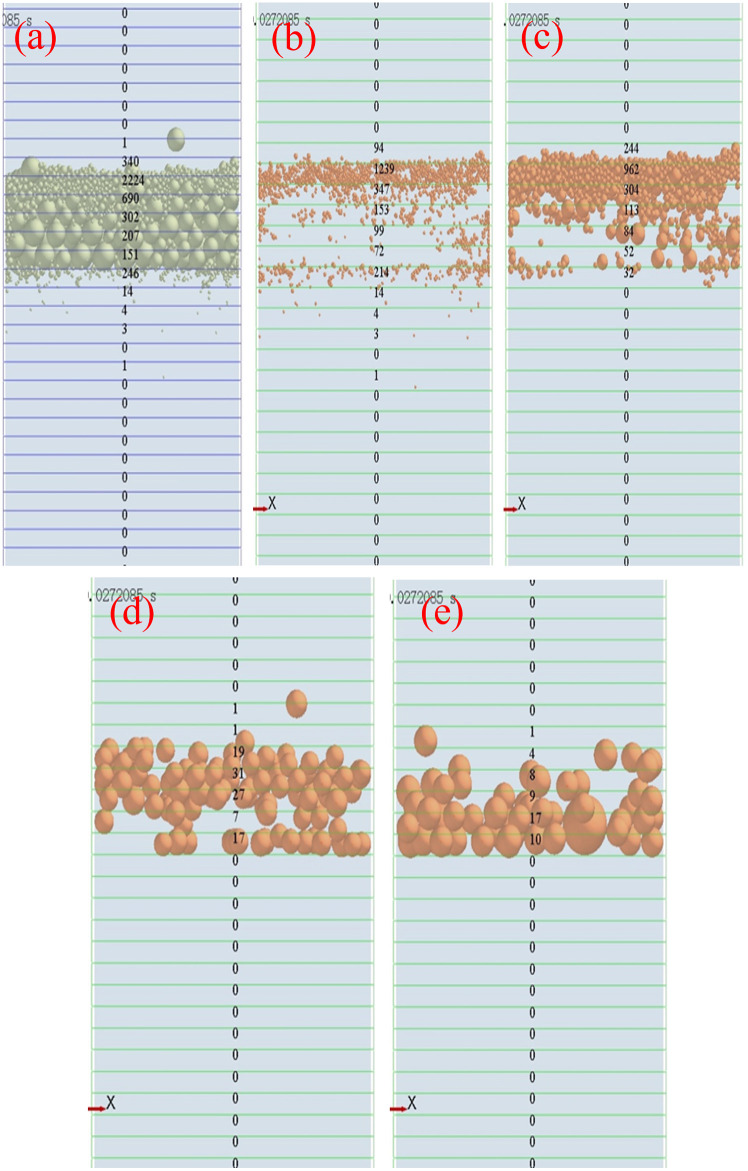
The results of particle migration under pressure of 0.5 MPa. (a) to (e) show the infiltration depths of the total particles and particles 1,2,3,4, respectively.

**Fig 7:**
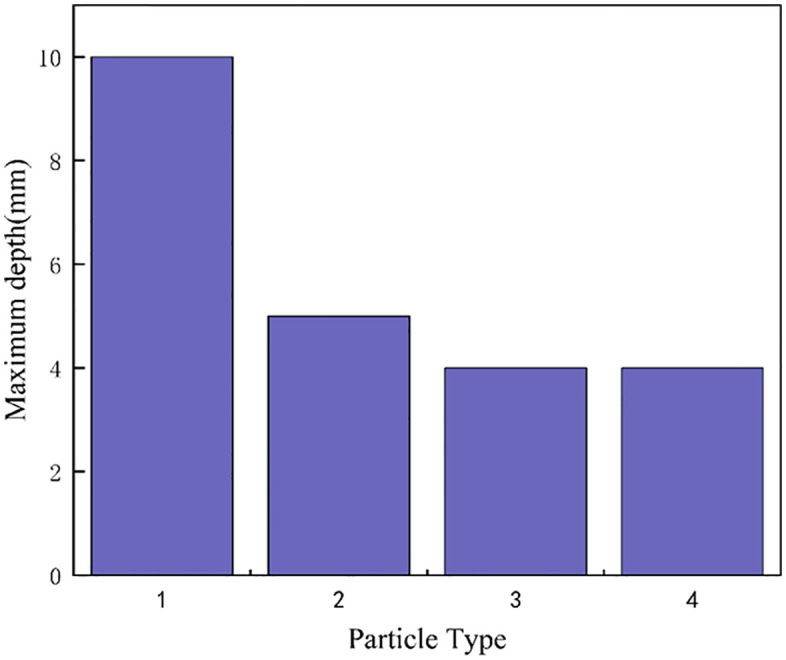
Depth of particle infiltration under pressure of 0.5 MPa.

The simulation results under the system pressure of 0.5 MPa are shown in Fig 6 and Fig 7.

In Fig 6, the values in the figure represent the number of particles in each layer, starting from 2224 in Fig 6a, and each layer below represents a depth of 1 mm. It can be clearly seen from the infiltration depth data that there are significant differences in the infiltration capacity of particles with different particle sizes. The infiltration depth of particle 1 reaches 10 mm, and the infiltration ability is the strongest among several particles. This is mainly because the particle size of particle 1 is small, the resistance is relatively small when moving in the fluid, and it can migrate more freely with the fluid in the fracture, so as to penetrate into the deeper position. The infiltration depth of particle 2 is 5 mm.Compared with particle 1, its infiltration capacity is obviously weakened. With the increase of particle size, the resistance of particles moving in the crack increases, and the collision and friction with the crack wall are enhanced, which leads to the decrease of its migration ability and more staying in the shallow layer. The infiltration depth of particles 3 and 4 is 4 mm, and the infiltration capacity of the two is similar and weak.

**Fig 8:**
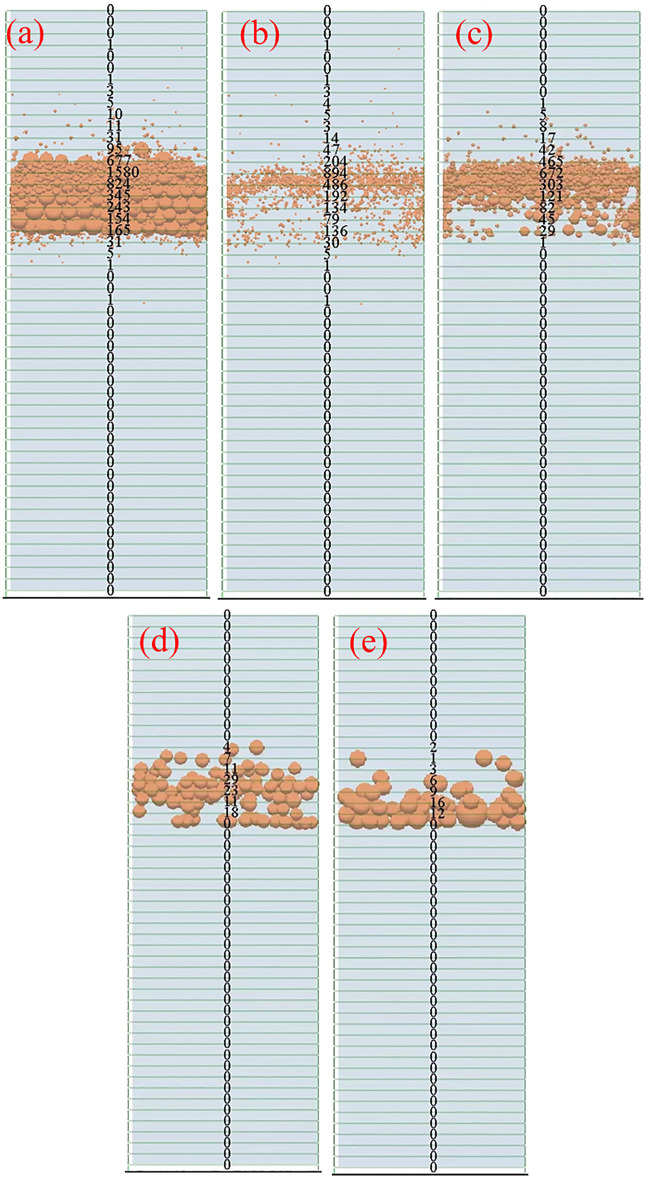
The results of particle migration under pressure of 1 MPa. (a) to (e) show the infiltration depths of the total particles and particles 1,2,3,4, respectively.

**Fig 9:**
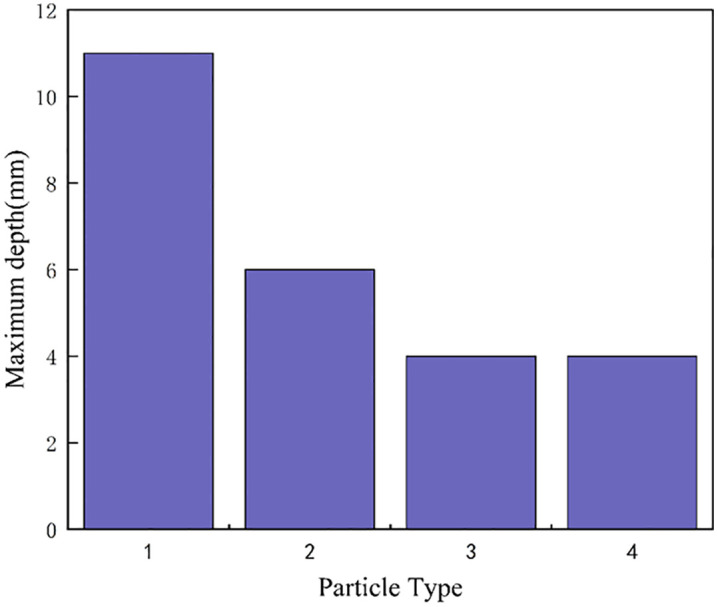
Depth of particle infiltration under pressure of 1 MPa.

The simulation results under the system pressure of 1MPa are shown in Fig 8 and Fig 9.

As can be seen from Fig 8 and Fig 9, starting from 1580 in Fig 8a, each layer below represents a depth of 1 mm. The infiltration depth of particle 1 is 11 mm, and the infiltration depth is deep. The infiltration depth of particle 2 is 6 mm, and its infiltration capacity is obviously weakened compared with particle 1. The infiltration depth of particles 3 and 4 is 4 mm.

#### 3.1.2. Simulation results under different temperatures.

**Fig 10:**
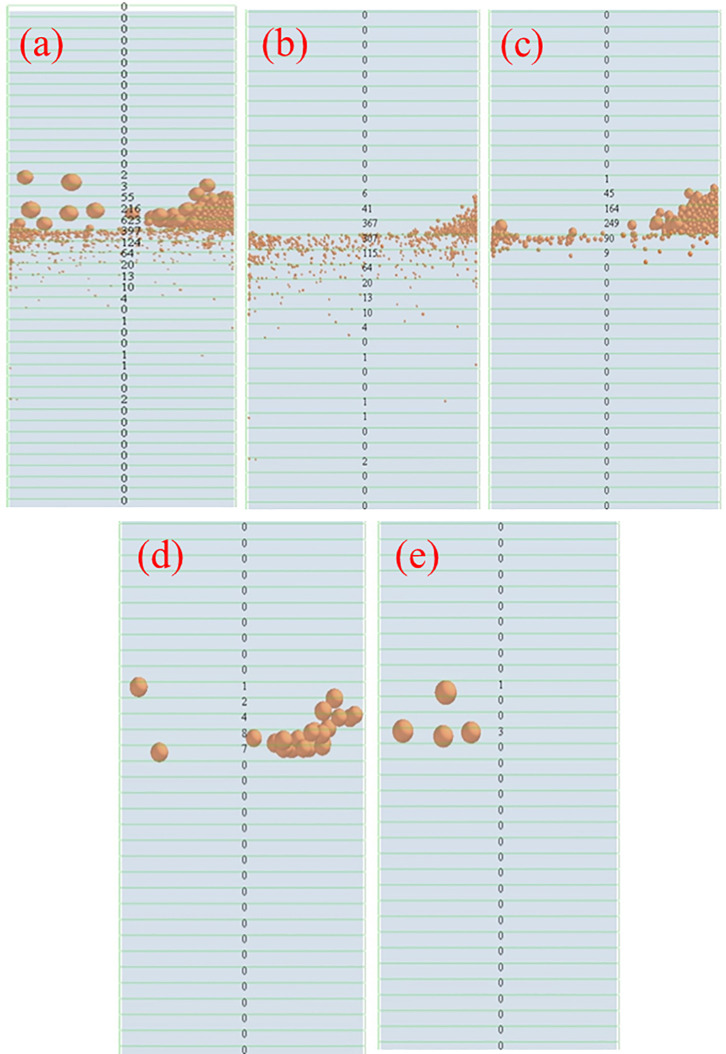
The results of particle migration at 60 °C. (a) to (e) show the infiltration depths of the total particles and particles 1,2,3,4, respectively.

**Fig 11:**
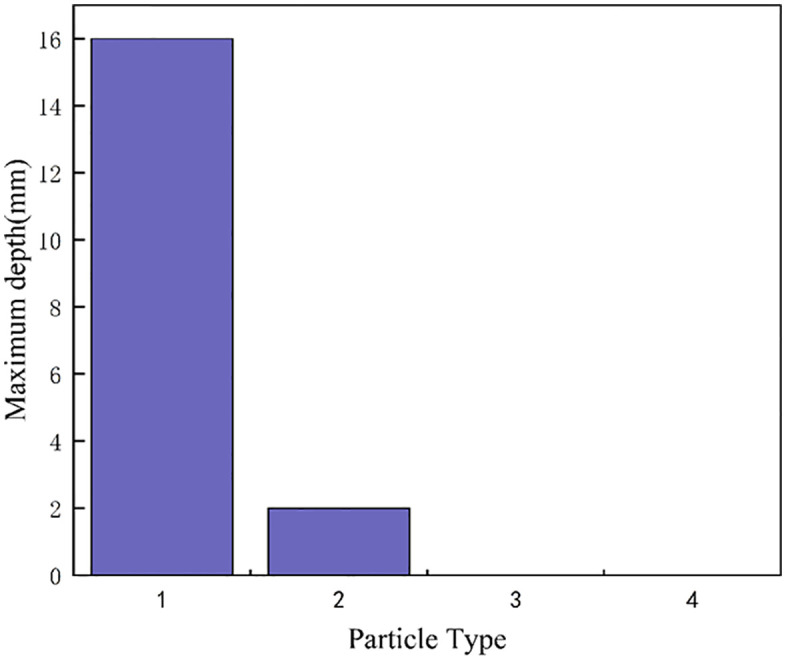
Depth of particle infiltration at 60 °C.

In order to systematically analyze the influence of temperature on the pollution behavior during well washing operation in sand-filled fractures, three temperature conditions of 60 °C, 80 °C and 100 °C were set up under the condition of fixed flow rate (1m/s). Based on the Vickers criterion, the particle size distribution was configured, and the migration and retention process of particles in fractures was simulated by the motion of solid particles. The numerical simulation of dynamic pollution was carried out to reveal the influence of temperature change on the pollution mechanism. The particle migration under the condition of 60 °C is shown in Fig 10 and Fig 11.

At 60 °C, starting from 623 in Fig 10a, each layer below represents a depth of 1 mm. The infiltration depth of particle 1 is 16 mm, while the infiltration depths of particles 2, 3 and 4 are 2 mm, 0 mm and 0 mm, respectively. Combined with the particle distribution characteristics shown in Fig 10, particle 1 has the smallest particle size and is obviously carried by the fluid, which can penetrate deep into the fracture, but it exists in a dispersed form in a deeper position, indicating that its plugging effect is weak. In contrast, particles 2, 3 and 4 are more likely to be detained near the entrance of the fracture due to the increase of particle size, especially when the infiltration depth of particles 3 and 4 is 0 mm, indicating that the particles of these two kinds of particle sizes are almost completely detained in the superficial area at 60 °C and accumulate on the surface. The larger particle size makes them face greater resistance when they move in the fluid, and it is difficult to overcome various obstacles in the cracks to penetrate deeply, and the infiltration is almost stopped in the shallow layer.

**Fig 12:**
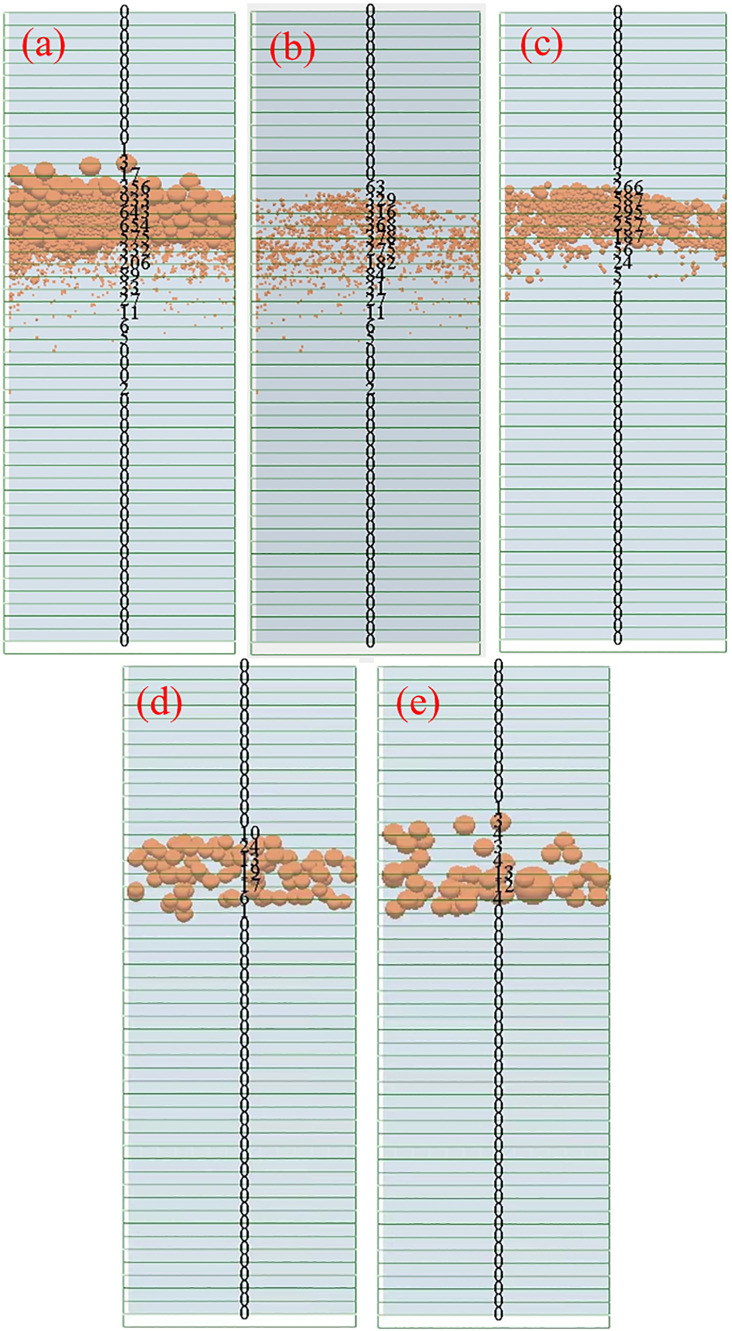
The results of particle migration at 80 °C. (a) to (e) show the infiltration depths of the total particles and particles 1,2,3,4, respectively.

**Fig 13:**
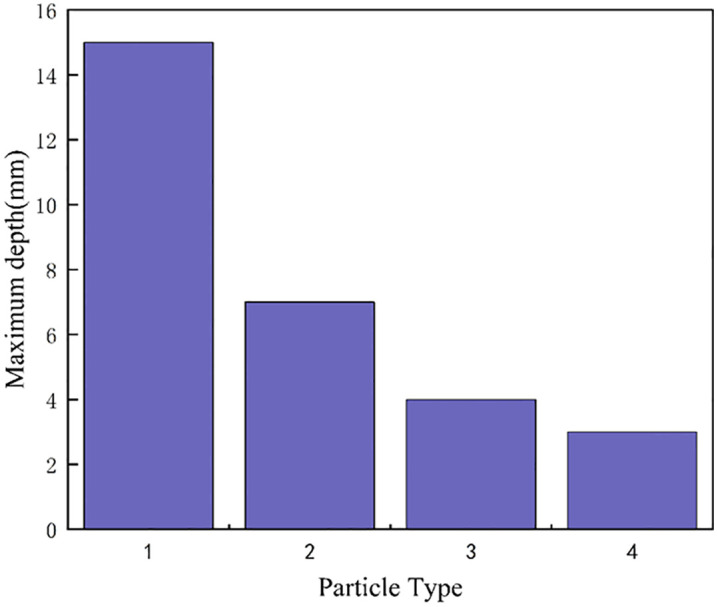
Depth of particle infiltration at 80 °C.

At the temperature of 80 °C, the migration simulation results of particles with different particle sizes in sand-filled fractures are shown in Fig 12 and Fig 13.

At 80 °C, starting from 933 in Fig 12a, each layer below represents a depth of 1 mm. The infiltration depths of particles 1, 2, 3 and 4 are 15 mm, 7 mm, 4 mm and 3 mm, respectively. Compared with the results at 60 °C, the overall infiltration depth of the particles at 80 °C decreases, indicating that the increase of temperature significantly enhances the retention ability of the particles at the near end of the fracture, which is conducive to the rapid formation of a dense plugging layer in the near-well area, thus effectively inhibiting the further leakage of fluid to the deep. The increase of temperature not only regulates the migration behavior of particles, but also optimizes the location distribution and structural stability of the plugging layer by promoting shallow dense accumulation.

**Fig 14:**
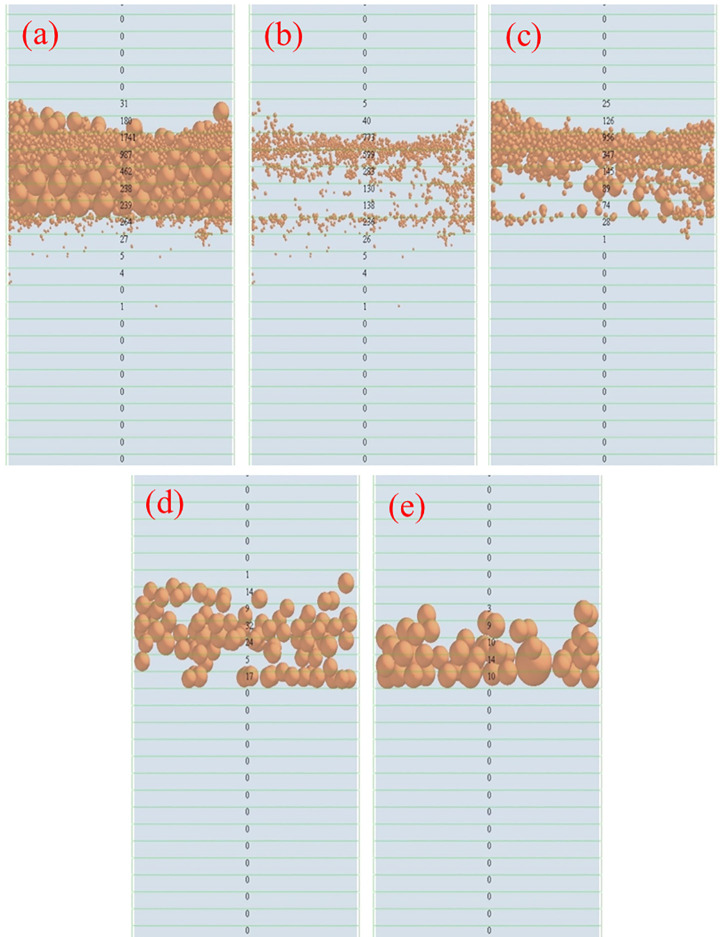
The results of particle migration at 100 °C. (a) to (e) show the infiltration depths of the total particles and particles 1,2,3,4, respectively.

**Fig 15:**
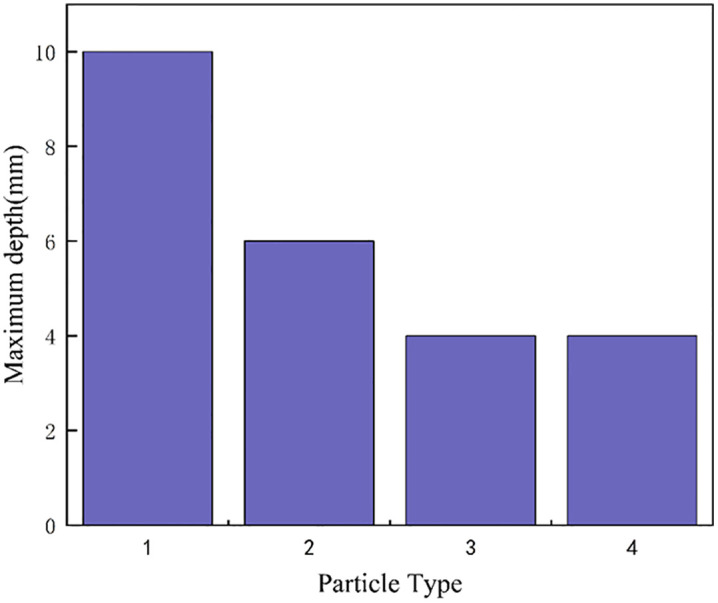
Depth of particle infiltration at 100 °C.

At a temperature of 100 °C, the migration simulation results of particles with different particle sizes in sand-filled fractures are shown in Fig 14 and Fig 15.

At 100 °C, starting from 1741 in Fig 14a, each layer below represents a depth of 1 mm. The infiltration depths of particles 1, 2, 3 and 4 are 10 mm, 6 mm, 4 mm and 4 mm, respectively, which are further reduced compared with those at 60 °C and 80 °C. The infiltration depth of particle 1 is only 10 mm, which is significantly lower than its value at lower temperature, indicating that the sealing ability of smaller particle size particles in the surface area is significantly enhanced under high temperature environment. The increase of temperature causes the thermal expansion of particles, especially for small-sized particles, which makes the volume slightly larger and the surface interaction stronger, so that it is more likely to collide and stay near the crack entrance in the early stage of migration, forming a tight shallow accumulation structure. Secondly, the high temperature leads to the decrease of the viscosity of the flushing fluid, which weakens its ability to suspend and carry particles, and promotes the deposition of particles earlier, especially in the near-well area to form an effective plugging layer [25]. These two factors work together to make the particle invasion depth shallower under high temperature conditions, and the position of the plugging layer is closer to the fracture inlet, thus effectively inhibiting the further leakage of the fluid to the deep.

### 3.2. Experimental results

#### 3.2.1. Rheological properties of well flushing fluid.

The results of the rheological properties of the flushing fluid are shown in Table 5. From the data of rheological properties, the apparent viscosity (AV) and plastic viscosity (PV) of the flushing fluid decrease with the increase of temperature. This is because the increase of temperature aggravates the thermal movement between fluid molecules, weakens the intermolecular force and the friction effect between particles, thus reducing the internal friction resistance of the system. The yield value (YP) decreases with the increase of temperature, indicating that the suspension capacity of the plugging agent decreases gradually in the range of 45 ~ 105 °C.

**Table 5:** Evaluation of rheological properties of well flushing fluid.

Temperature(℃)	AV(mPa·s)	PV(mPa·s)	YP(Pa)
45	22.5	12	5.3655
65	18.5	8	5.3655
85	18	8	5.11
105	16.5	7	4.8545

#### 3.2.2. Pollution behavior under different pressures.

**Fig 16:**
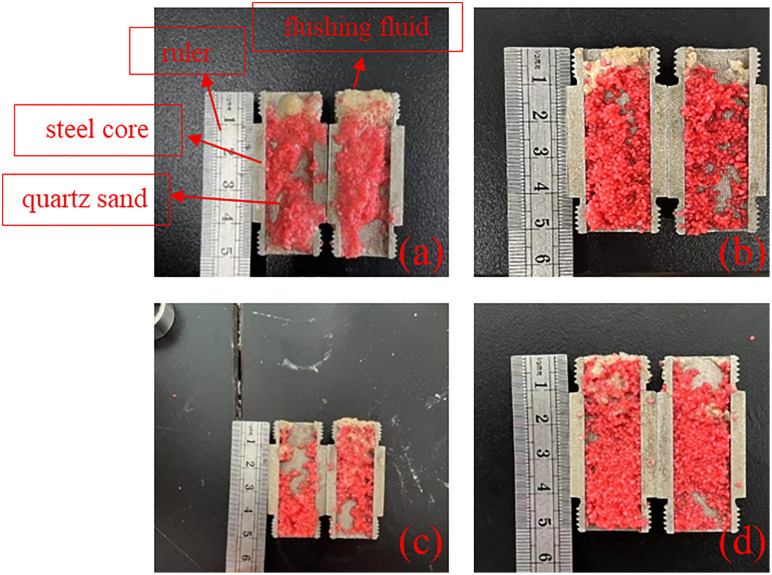
Plugging effective area under different displacement pressure. (a) to (d) illustrate the particle invasion depths under displacement pressures of 2 MPa, 3 MPa, 4 MPa, and 5 MPa, respectively.

In order to study the effect of displacement pressure on the plugging effect, four groups of experiments were carried out under the conditions of 2,3,4,5MPa respectively. The results are shown in Table 6 and Fig 16.

**Table 6:** Plugging experimental results under different displacement pressures.

Displacement pressure/MPa	2	3	4	5
Maximum Depth /mm	9	12	14	16

According to the results of plugging experiments under different displacement pressures in Table 6, as the displacement pressure increases from 2 MPa to 5 MPa, the depth of particle intrusion gradually increases from 9 mm to 16 mm. This shows that the displacement pressure is positively correlated with the invasion depth, that is, the greater the displacement pressure, the deeper the invasion depth of the particles in the medium; on the contrary, the smaller the displacement pressure, the shallower the invasion depth, the more conducive to the rapid formation of a dense plugging layer in the shallow layer. In particular, when the displacement pressure is 2 MPa, the invasion depth reaches the minimum value of 9 mm. At this time, the temporary plugging agent particles can accumulate rapidly in the shallow layer and form a tight plugging layer, which can effectively inhibit the further invasion of the fluid and obtain the optimal plugging effect.

#### 3.2.3. Pollution behavior under different temperatures.

**Fig 17:**
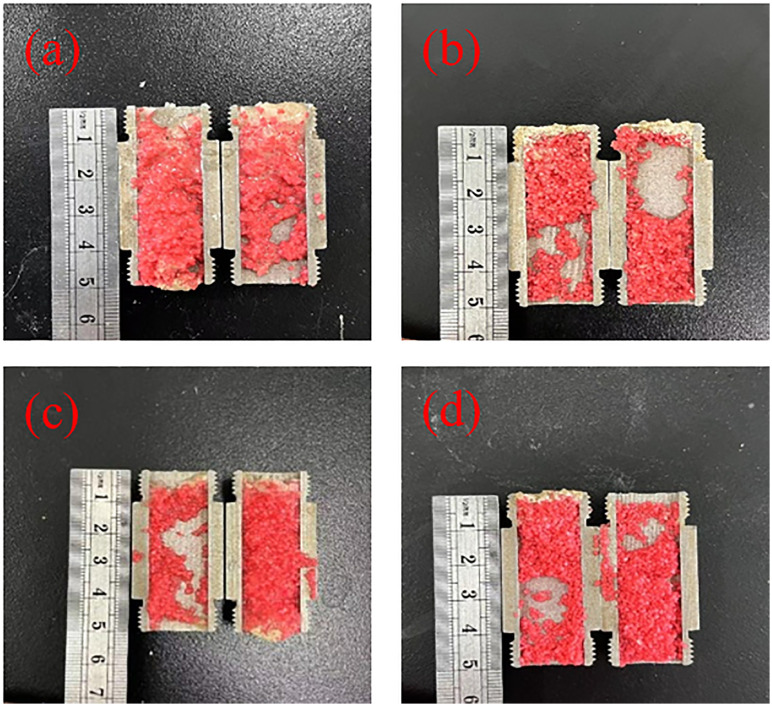
Plugging effective area at different temperatures. (a) to (d) illustrate the particle invasion depths under displacement pressures of 45℃, 65℃, 85℃ and 105℃, respectively.

In order to study the influence of different temperatures on the plugging effect, four groups of experiments were carried out at 45–105 °C. The results are shown in Table 7 and Fig 17.

**Table 7:** Experimental results of plugging at different temperatures.

Temperature /℃	45	65	85	105
Maximum Depth /mm	10	9	6	2

As the temperature increases from 45 °C to 105 °C, the invasion depth of the temporary plugging material continues to decrease from 10 mm to 2 mm, indicating that the retention of the material on the core surface is enhanced under high temperature environment, forming a more effective shallow plugging. As the temperature increases, the depth of intrusion becomes shallow. On the one hand, the thermal expansion and contraction of the plugging particles increase the volume of the particles, increase the volume of the particles in the cracks, and form a more effective accumulation. On the other hand, the suspension of the treatment agent in the well flushing fluid is reduced under high temperature conditions, resulting in the accumulation of particles on the surface. This is consistent with the results of Yang Xianyu et al [26].

### 3.3. Discussion

**Fig 18:**
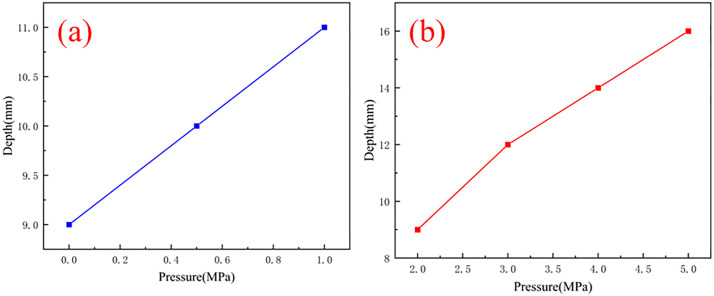
Invasion depth of particles under different pressures. (a) shows the simulation results. (b) shows the experimental results.

**Fig 19:**
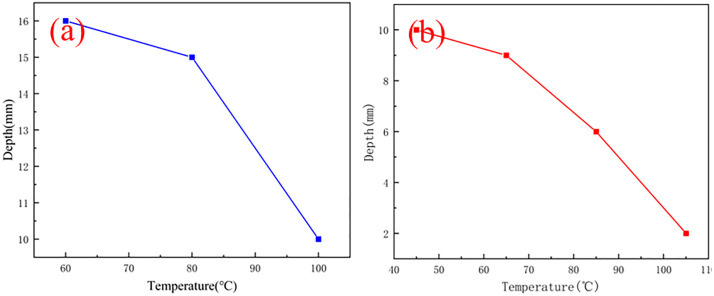
Invasion depth of particles at different temperatures. (a) shows the simulation results. (b) shows the experimental results.

Fig 18 and Fig 19 systematically compare the numerical simulation and indoor experimental results of particle invasion depth in sand-filled cracks with pressure and temperature changes.

In Fig 18 (particle invasion depth under different pressure conditions), the simulation results show that when the pressure increases from 0 MPa to 1.0 MPa, the invasion depth gradually increases from 9.0 mm to 11.0 mm. In the experimental results, the invasion depth increased from 9 mm to 16 mm when the pressure increased from 2 MPa to 5 MPa, and the two trends remained consistent. The increase of the displacement pressure difference will significantly increase the energy carried by the fluid to the particles, prompting the particles to overcome the resistance to migrate to the deep part of the fracture, resulting in an increase in the depth of invasion and an increase in the risk of fluid leakage.

In Fig 19, the simulation results show that when the temperature rises from 60 °C to 100 °C, the invasion depth decreases from 16 mm to 10 mm; in the experimental results, the intrusion depth decreases from 10 mm to 2 mm when the temperature increases from 45 °C to 105 °C. Both simulation and experiment show that the intrusion depth decreases significantly with the increase of temperature. On the one hand, the increase of temperature will cause the thermal expansion of the plugging particles, especially the increase of the volume of the larger particles and the enhancement of the surface interaction, so that it is easier to stay in the shallow layer and form a dense accumulation. On the other hand, the high temperature will reduce the viscosity of the well flushing fluid, weaken its suspension and sand carrying capacity, and make the particles deposit in the near well area earlier. Under the synergistic effect of the two, the high temperature condition promotes the rapid formation of the plugging layer in the shallow part of the fracture, which effectively inhibits the further leakage of the fluid to the deep part.

In summary, both experimental and simulation results exhibit highly consistent patterns of change under both pressure and temperature conditions. This not only validates the reliability of the numerical model but also reveals, from a mechanistic perspective, the key controlling factors of contamination behavior in sand-packed fractures under complex conditions. Pressure primarily affects the intrusion depth by enhancing the sand-carrying capacity, while temperature alters the sealing location by regulating particle-fluid interactions. This study provides theoretical guidance for on-site well-washing operations: under high-pressure conditions, particle gradation and injection parameters should be optimized to control deep contamination; under high-temperature conditions, the temperature effect can be fully utilized to achieve efficient shallow sealing through the selection of a suitable particle system, thereby enhancing the pertinence and effectiveness of well-washing operations under complex conditions.

## 4. Field test and performance evaluation

### 4.1. Overview of the field case

Xuping X well is a horizontal well drilled in Zhuang 138 well area in 2018. The buried depth (inclined depth) of the reservoir is 1866.0m, and the buried depth (vertical depth) of the reservoir is 1654.01m. The horizontal section is 1289 m long, the drilling rate is 61.44%, 581 m oil layer + 211 m poor oil layer. On September 5,2019, the casing bridge plug fracturing was used to complete the test, and a total of 22 segments were fractured. The total sand volume is 1220.0 m^3^, the average sand ratio is 6.25%, the displacement is 6.0–12.0 m^3^ / min, the total liquid volume into the ground is 22859.0 m^3^, and there is no swabbing. In the early stage of the well, the daily liquid production was 8.55 m^3^, the daily oil production was 6.52 t, the water cut was 23.7%, and the dynamic liquid level was 983 m. The well has no measures over the years; before the measures, the daily liquid production is 2.35 m^3^, the daily oil production is 1.98 t, the water content is 15.7%, the dynamic liquid level is 1124 m, the cumulative oil production is 4645 t, the cumulative water production is 4245 m^3^, and the flowback rate is 18.6%.

### 4.2. Temporary plugging sand washing process

#### 4.2.1. Preliminary preparation.

(1) A total of 200 m³ of clean water was prepared on the construction site.
(2) The required equipment included one set of Φ114 mm integrated sand washing and scraping tool, one set of KQ65/35 type high-pressure wellhead, 1300 m of Φ73 mm chamfered tubing, 2000 m of Φ89 mm chamfered tubing, 1 mixing tank with a volume of 20–40 m³, one sand settling tank with a volume of 40–70 m³, and one mixing tank with a volume of 3–5 m³.
(3) Two sets of Model 1050 pump trucks were deployed, which were required to be in good operating condition with complete pipelines and accessories. The displacement of a single unit should be no less than 1000 L/min.
(4) Equipment requirement: The hoisting system and derrick should have a load-bearing capacity of no less than 50 tons.
(5) Common tools, fire-fighting equipment, and labor protection articles were fully prepared in accordance with the requirements of normal construction operations.
(6) The formula of the temporary plugging sand washing fluid was configured as follows: 0.3% HPAM + 6% HXC + 23% CY-2 + 45% WB-5 + 26% CP-2. The formula ratio was adjusted in real time according to the variation of wellbore leakage rate during the sand washing process.

#### 4.2.2. Oil displacement and well killing.

(1) Clean water was used for oil displacement and well killing. The oil displacement pipelines and blowdown pipelines were connected and fixed firmly, and a pressure test was conducted on the oil displacement lines with clean water. The test pressure was not less than 1.5 times the maximum designed construction pressure, and the pressure was maintained for 5 minutes without leakage, which was regarded as qualified.
(2) Blowdown was controlled, and oil and gas were displaced by reverse circulation with clean water at a displacement of 100–200 L/min.
(3) The balance between inlet and outlet flow was maintained, with the displacement controlled at 300–500 L/min. Circulation was continued until the properties of the inlet and outlet fluids were basically consistent, and pumping was not allowed to stop midway.
(4) After 30 minutes of observation, the reverse circulation oil displacement operation was completed when no overflow was observed at both the inlet and outlet.

#### 4.2.3. Pulling out the original well string.

The original production well string was pulled out, inspected, and its data was verified and cleaned thoroughly by washing.

#### 4.2.4. Temporary plugging, sand washing and well flushing.

(1) A Φ114 mm integrated sand washing and scraping tool combined with Φ73 mm chamfered tubing was used to accurately detect the sand surface at a depth of 1695.78 m.
(2) After the sand surface was detected, the sand washing string was lifted to 1668 m for an injection test to determine water absorption capacity, and the result showed complete lost circulation.
(3) On September 12, temporary plugging agent was prepared on site, and wellbore temporary plugging construction was carried out. At 15:00, 6 m³ of gel was positively injected into the well at a pumping displacement of 500 L/min and a pump pressure of 0 MPa. At 19:00, a total of 41 m³ of temporary plugging agent was prepared and positively squeezed into the well, with the displacement adjusted from 400 L/min to 300 L/min and the pressure rising to 5 MPa. At 20:00, 5 m³ of gel was prepared and positively squeezed into the well at a displacement of 300–200 L/min, with the pumping pressure reaching 9.3 MPa and the shut-in pressure being 8.7 MPa. After maintaining the pressure for 10 minutes, the pressure dropped to 4.6 MPa, indicating that the temporary plugging was qualified.
(4) On September 13, construction was suspended due to the rectification of potential safety hazards.
(5) On September 14, sand washing fluid was prepared for well flushing and sand washing. Pumping was started for circulating flushing at 11:04 with a displacement of approximately 500 L/min, and gas and crude oil were returned from the outlet; normal outlet return was achieved at 11:09. A total leakage of about 2.5 m³ occurred during well shut-in at night. A small amount of gel and 6 m³ of temporary plugging agent were returned at 11:20. The pipeline process was switched at 11:40 to conduct reverse sand washing. From 13:00–19:30, reverse sand washing was performed to a well depth of 2085.89 m, with a total footage of 309.11 m. The sand washing interval was 1695.78 ~ 2085.89 m, with a total sand washing time of 4.17 hours. Excluding the 2.5 m³ leakage during night shut-in, the total leakage during the sand washing process was 3.51 m³, and the leakage rate per unit time was 0.84 m³/h.
(6) On September 15, the drilling string was run to 2085.89 m, and pumping was started for circulation at 8:04 in preparation for sand washing, with a displacement of approximately 900 L/min; normal outlet return appeared at 8:14. From 8:30–18:40, reverse sand washing was conducted to a well depth of 2731.96 m, with a total footage of 646.07 m. Part of the tubing was pulled out after circulating flushing. The total sand washing time was 10.83 hours, and the leakage rate per unit time was 0.82 m³/h.
(7) On September 16, the drilling string was run to 2731.96 m, and pumping was started for circulation at 10:05 with a displacement of approximately 800 L/min; stable return was achieved at 10:10. Reverse flushing and sand washing were carried out from 10:30–11:40, and the drilling string was pulled out when no further footage was obtained. The total sand washing time was 1.17 hours, and the leakage rate per unit time was 1.7 m³/h.
(8) On September 17, power drilling tools were run to 2731.96 m. Fluid was injected into the wellbore at 15:14 with a displacement of 500 ~ 700 L/min, and fluid return was observed at 15:28. After normal circulation was established, clean water was used for sand washing, with a leakage rate per unit time of 5.24 m³/h.

**Fig 20:**
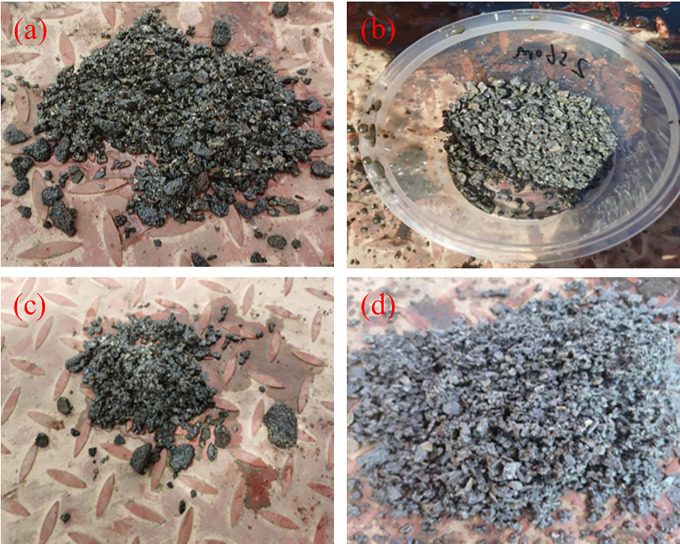
The wellbore scale sample returned during the sand washing process. (a) show the scale samples at 2085.89m. (b) show the scale samples at 2260m. (c) show the scale samples at 2731.96m. (d) show the scale samples at 3137m.

Fig 20 shows the scale sample returned in the sand washing process, and the maximum particle size of the scale sample is 2.5 cm, indicating that the workover fluid has a strong ability to carry solid particles, which not only realizes the temporary plugging and leakage reduction in the sand washing process, but also significantly improves the operation efficiency of sand washing and descaling.

### 4.3. Field performance

(1) Effect of temporary plugging and leakage reduction

By using temporary plugging sand washing workover fluid, the technical problem of loss of return leakage before operation of Xuping X well is effectively solved, the normal circulation of wellbore before sand washing operation is realized, and the safety and efficiency of sand washing operation process are guaranteed.

**Fig 21:**
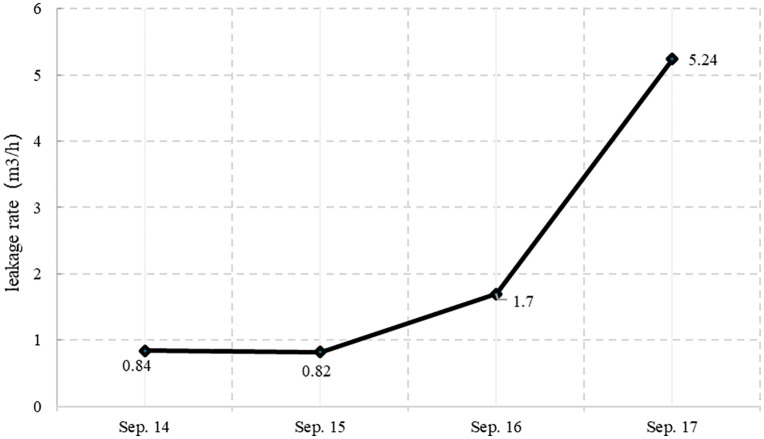
Statistics on Leakage Rate of Well Xuping X.

As shown in Fig 21, when the clean water is used for sand washing, the leakage per unit time is 5.24m^3^.When the X well is used for sand washing, the leakage per unit time is 0.84 ~ 1.7m^3^, with an average value of 1.27m^3^, and the leakage per unit time is reduced by 75.8%.

(2) Production recovery of Xuping X well

**Fig 22:**
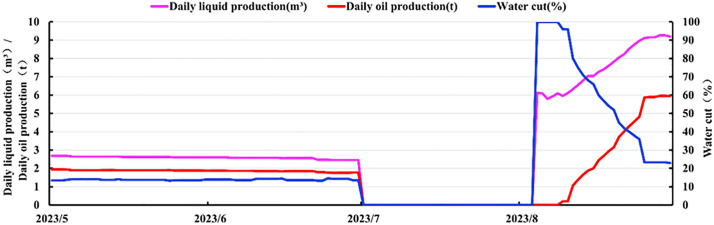
Comparison of production before and after production operation in Xuping x well.

As shown in Fig 22, after the well was put into production, oil began to appear in 5 days. After 22 days, the water content decreased from 100% to 26%, and the daily oil production reached 5.95 t. It is considered that the temporary plugging sand washing workover fluid system used in this project has a low degree of damage to the reservoir, which is very conducive to the recovery of production after operation. Taking the daily oil production before and after operation as the evaluation index to measure the degree of reservoir damage, the daily oil production of Xuping X well increased by 3.5 t. It is considered that the damage rate of workover fluid to reservoir is low when the well is sand washed.

## 5. Conclusions

In this study, the sand filling fracture was taken as the research object. On the basis of completing the filling operation of quartz sand in the fracture area, the fracture pore structure was reconstructed by CT technology, and the Vickers criterion was selected as the theoretical basis for the optimization of particle system. By combining CFD-DEM numerical simulation with indoor dynamic pollution experiments, the influence of pressure and temperature on particle migration behavior and sealing effectiveness was systematically analyzed, revealing the formation law of sand-packed fracture pollution under complex working conditions. The main conclusions are as follows:

(1) The high pressure environment will significantly aggravate the pollution degree of the filling area. With the increase of displacement pressure, the depth of particle invasion gradually increases, indicating that the increase of pressure difference will enhance the sand carrying capacity of fluid, promote the migration of particles to the deep part of fractures, lead to the uneven distribution of plugging layers, and significantly increase the risk of deep pollution.
(2) High temperature conditions can effectively inhibit the deep invasion of particles and optimize the shallow plugging effect. As the temperature increases, the depth of particle intrusion decreases significantly. On the one hand, the thermal expansion and surface interaction of particles are enhanced, which promotes the retention of large particles in the shallow layer to form dense accumulation. On the other hand, the decrease of the viscosity of the well flushing fluid weakens the suspension ability, so that the particles are deposited in the near well area earlier, thus forming an efficient shallow plugging layer.
(3) The numerical simulation and experimental results are highly consistent in the trend of pressure and temperature, which verifies the reliability of CFD-DEM model in the simulation of complex working conditions.
(4) The field test results show that optimizing the washing fluid suitable for the formation temperature and reasonably controlling the construction displacement can reduce the pollution degree in the sand washing process and shorten the recovery time of single well production.
